# Partial exogastrulation due to apical–basal polarity of F‐actin distribution disruption in sea urchin embryo by omeprazole

**DOI:** 10.1111/gtc.12934

**Published:** 2022-04-09

**Authors:** Kaichi Watanabe, Yuhei Yasui, Yuta Kurose, Masashi Fujii, Takashi Yamamoto, Naoaki Sakamoto, Akinori Awazu

**Affiliations:** ^1^ Graduate School of Integrated Sciences for Life Hiroshima University Higashi‐Hiroshima Japan; ^2^ Department of Mathematical and Life Sciences Graduate School of Science, Hiroshima University Higashi‐Hiroshima Japan; ^3^ Research Center for the Mathematics on Chromatin Live Dynamics Hiroshima University Higashi‐Hiroshima Japan

**Keywords:** actin, gastrulation, omeprazole, partial exogastrulation, sea urchin embryo

## Abstract

Gastrulation is a universal process in the morphogenesis of many animal embryos. Although morphological and molecular events in gastrulation have been well studied, the mechanical driving forces and underlying regulatory mechanisms are not fully understood. Here, we investigated the gastrulation of embryos of a sea urchin, *Hemicentrotus pulcherrimus*, which involves the invagination of a single‐layered vegetal plate into the blastocoel. We observed that omeprazole, a proton pump inhibitor capable of perturbing the left–right asymmetry of sea urchin embryo, induced “partial exogastrulation” where the secondary invagination proceeds outward. During early gastrulation, intracellular apical–basal polarity of F‐actin distribution in vegetal half was higher than those in animal half, while omeprazole treatment disturbed the apical–basal polarity of F‐actin distribution in vegetal half. Furthermore, gastrulation stopped and even partial exogastrulation did not occur when F‐actin polymerization or degradation in whole embryo was partially inhibited via *RhoA* or *YAP1* knockout. A mathematical model of the early gastrulation reproduced the shapes of both normal and exogastrulating embryos using cell‐dependent cytoskeletal features based on F‐actin. Additionally, such cell position‐dependent intracellular F‐actin distributions might be regulated by intracellular pH distributions. Therefore, apical–basal polarity of F‐actin distribution disrupted by omeprazole may induce the partial exogastrulation via anomalous secondary invagination.

## 1 INTRODUCTION

Gastrulation is an essential morphogenic process in various animals, wherein a ball of single‐layered cells (blastula) differentiates into a multilayered gastrula or early embryo (Nájera & Weijer, 2020; Shindo, 2018; Stower & Bertocchini, 2017). Species‐specific variations in this process provide the basis for particular animal morphologies and lead to the generation of internal organs. The basic mechanism of whole embryonic transformations induced by organizational rearrangement and cell movements is evolutionarily conserved.

Sea urchin embryos are commonly used for studying various morphogenetic processes universally observed in animals, such as the left–right symmetry breakage (Aihara & Amemiya, 2001; McCain & McClay, 1994; Takemoto et al., 2016; Warner & McClay, 2014), neural network formations (Burke et al., 2014; McClay et al., 2018; Yaguchi et al., 2010), and gastrulation (Dan & Okazaki, 1956; Gustafson & Kinnander, 1960; Hardin & Cheng, 1986; Kominami & Takata, 2004; Martik & McClay, 2017) due to its evolutionary position as diverged at early period of deuterostome evolution. Furthermore, the gene regulatory network controlling endomesoderm specification in sea urchin embryos have been well studied (Davidson et al., 2002; Oliveri & Davidson, 2004). Sea urchin gastrulation progresses through the following five steps (Kominami & Takata, 2004): Step 1 (after hatching), the embryo becomes an epithelial monolayer with a thickened vegetal plate; step 2 (primary invagination), the vegetal plate bends to invade the blastocoel and a short tubular archenteron is formed; step 3 (lag phase in archenteron elongation), secondary mesenchyme cells (SMCs) appear at the tip; step 4 (secondary invagination), the archenteron elongates by pulling on the filopodia of the SMC and cell rearrangement; step 5 (tertiary invagination), presumptive endodermal cells are recruited into the archenteron. Steps 1 and 2 have been conventionally referred to the primary invagination, and steps 3 and 4 to the secondary invagination (Dan & Okazaki, 1956; Gustafson & Kinnander, 1956).

Embryos of some sea urchin species exhibit anomalous morphogenesis (exogastrulation) under various treatments where the vegetal plate of the embryo prolapses outward instead of invaginating. For example, the archenteron of embryos cultured in the presence of LiCl or sugar is completely evaginated due to the depolymerization of SMC microtubules in the blastocoel space connecting the primary intestine and ectoderm (Dan & Okazaki, 1956; Hardin & Cheng, 1986; Khurrum et al., 2004). These studies suggest that lifting the primary intestine from the vegetal plate by microtubules plays an important role in step 4 of gastrulation. Exogastrulation was also observed via Rab35 knockdown, which was also suggested to disturb whole embryonic cytoskeleton distribution (Remsburg et al., 2021).

Moreover, the vegetal plate bending in step 2 of normally cultured embryos of the sea urchin, *Lytechinus pictus*, occurs even when it is surgically isolated (Ettensohn, 1984). It is suggested that the cells at the archenteron tip should be bottle‐shaped (Nakajima & Burke, 1996), and those around the vegetal plate should be wedge‐shaped (Burke et al., 1991) to initiate primary invagination (Kominami & Takata, 2004). Additionally, mathematical models of vegetal poles suggest that primary invagination occurs under the appropriate force conditions for “apical constriction,” “cell tractor,” “apical contractile ring,” “apicobasal contraction,” and “gel swelling” (Davidson et al., 1995; Odell et al., 1981). However, it is unclear which of these effects are essential for development after primary invagination (steps 2).

In this study, the roles of the dominant factors in steps 2 and after of sea urchin gastrulation are determined through experimental analysis and mathematical modeling. First, omeprazole that was known as the gastric acid suppressant inhibiting the proton pump activity and as to perturbs left–right asymmetry of sea urchin embryo (Hibino et al., 2006) caused “partial” exogastrulation without the loss of microtubules in the blastocoel space of *Hemicentrotus pulcherrimus* (HP) embryos. Second, the intracellular apical–basal polarity of F‐actin distribution in vegetal side cells was stronger than those in animal side cells during the early gastrulation stage (steps 1 and 2), but omeprazole disturbed the polarities in vegetal side. Therefore, the partial exogastrulation was likely due to anomalous cytoskeletal behaviors. Third, clustered regularly interspaced short palindromic repeat (CRISPR) ‐ CRISPR‐associated nuclease 9 (Cas9)‐mediated knockout of cytoskeleton‐related genes was performed in HP embryos, and the apical–basal ratio of intracellular pH was analyzed using fluorescence imaging. Finally, simulations of morphogenic processes of normal and omeprazole‐treated (exhibiting exogastrulation) HP embryos during the pre‐early gastrulation were performed using a mathematical model utilizing the cytoskeletal force parameters determined using the above experiments.

## 2 RESULTS

### 2.1 Omeprazole treatment of HP embryos caused partial exogastrulation

**FIGURE 1:**
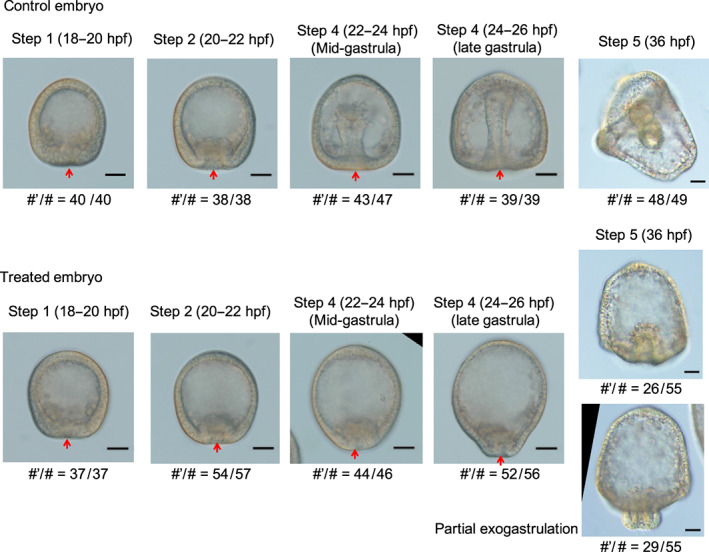
Developmental stages of control and omeprazole‐treated *Hemicentrotus pulcherrimus* (HP) embryos. Typical bright‐field images of the gastrulation process (steps 1, 2, 4, and 5) of sea urchin embryos (scale bars: 30 μm). Red arrows indicate vegetal pole positions. Gastrulation did not progress after step 2 in the treated embryos, and the outward protrusion of the vegetal pole side was more pronounced at step 4. In step 5, gastrulation stopped incompletely without penetrating the archenteron, and “partial” exogastrulation was observed in more than half of treated embryos (bellow image). # and #’ refer to the total number of sampled embryos and the number of embryos with similar shape to the image, respectively

The HP embryos exposed to omeprazole from immediately after fertilization and through development exhibited anomalous morphogenesis with partial exogastrulation (Figure 1). Detailed comparisons of the structural features around the vegetal pole between the normal (control) and treated embryos at each gastrulation step were performed. The following steps were designated based on those defined in a recent report (Kominami & Takata, 2004). In this case, the original step 3 is not shown because step 3 was obtained at 21–23 h post fertilization (hpf) that overlaps step 2 (20–22hpf) and step 4 (mid‐gastrula: 22–24 hpf).

**FIGURE 2:**
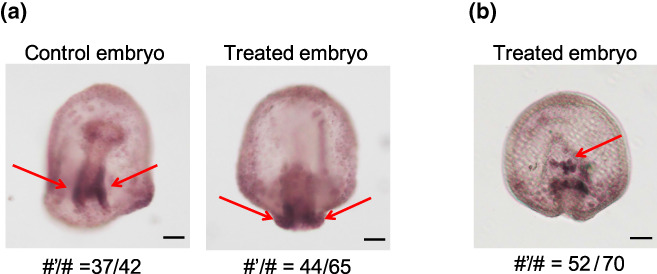
Visualization of endodermal tissue and secondary mesenchyme cells (SMCs). (a) Endodermal tissue was stained for endogenous alkaline phosphatase (red arrows) in control (left) and treated embryos (right) in step 5, where stained part located inner region in control embryos while stained located outer region in treated embryos (scale bars: 30 μm). (b) Typical locations SMCs in treated embryo in step 4 where *gcm*, the marker of SMC, was stained (red arrow) (scale bars: 30 μm). # and #’ refer to the total number of sampled embryos and the number of embryos clearly stained similar to the image, respectively

In step 1, control and treated embryos exhibited equivalent vegetal pole thickening (18–20 hpf). In step 2, primary invagination occurred in both control and treated embryos at the same time. However, intestinal invagination was slightly shallower in the treated embryos than that in the control embryos (20–22 hpf). In the mid‐gastrula stage (step 4; secondary invagination stage), treated embryos did not show further elongation of the archenteron into the blastocoel, and the shape of treated embryos elongated along the animal–vegetal axis (22–24 hpf). Meanwhile, in the control embryos, the archenteron was further elongated, and the presence of SMCs was obvious. In the late gastrula stage (step 4; secondary invagination stage), the outward protrusion of the vegetal pole side was pronounced in the treated embryo (24–26 hpf). The treated embryo had a comparatively little elongation of the archenteron into the blastocoel compared to the control embryos. In step 5 (36 hpf), gastrulation was arrested without penetrating the archenteron in the treated embryos. More than half of treated embryos exhibited “partial” exogastrulation, in which the tip of the archenteron folded inward due to normal primary invagination, but the remaining part extended outward during the secondary invagination. Here, the bottom half of archenteron was stained for endogenous alkaline phosphatase activity in control embryos (Figure 2a). On the other hand, in treated embryos showing partial exogastrulation, the part extending outward was stained (Figure 2a), indicating that the part corresponding to endodermal tissue was formed at the outer of these embryos. However, a part of the endodermal tissue was invaginated inward, suggesting that exogastrulated endodermal tissue may be lifted inward in the treated embryos after the secondary invagination.

During the secondary invagination phase, SMCs are detached from the tip of the archenteron and form contractile units; the connection between the archenteron tip and the animal pole tissue via the SMC and pseudopodia facilitates the traction of the archenteron (Dan & Okazaki, 1956; Hardin, 1988). In both control and treated embryos, the migration of SMCs from the tip of archenteron into the blastocoel was observed (Figure 2b), indicating that the SMCs migrated into the blastocoel of the treated embryo between steps 2 and 4 and pseudopodia were formed in the blastocoel.

Previously reported exogastrulation was induced by the loss of pseudopodia (Dan & Okazaki, 1956; Hoshi, 1979; Khurrum et al., 2004); therefore, partial exogastrulation in this report was expected to occur through a different mechanism. On the other hand, whole mount in situ hybridization showed the release of SMCs from archenteron in the treated embryos (Figure 2b). This result suggested that further lift of the archenteron into the blastocoel was induced by SMCs in the treated embryos.

### 2.2 Omeprazole perturbed cytoskeletal distributions in vegetal side cells

**FIGURE 3:**
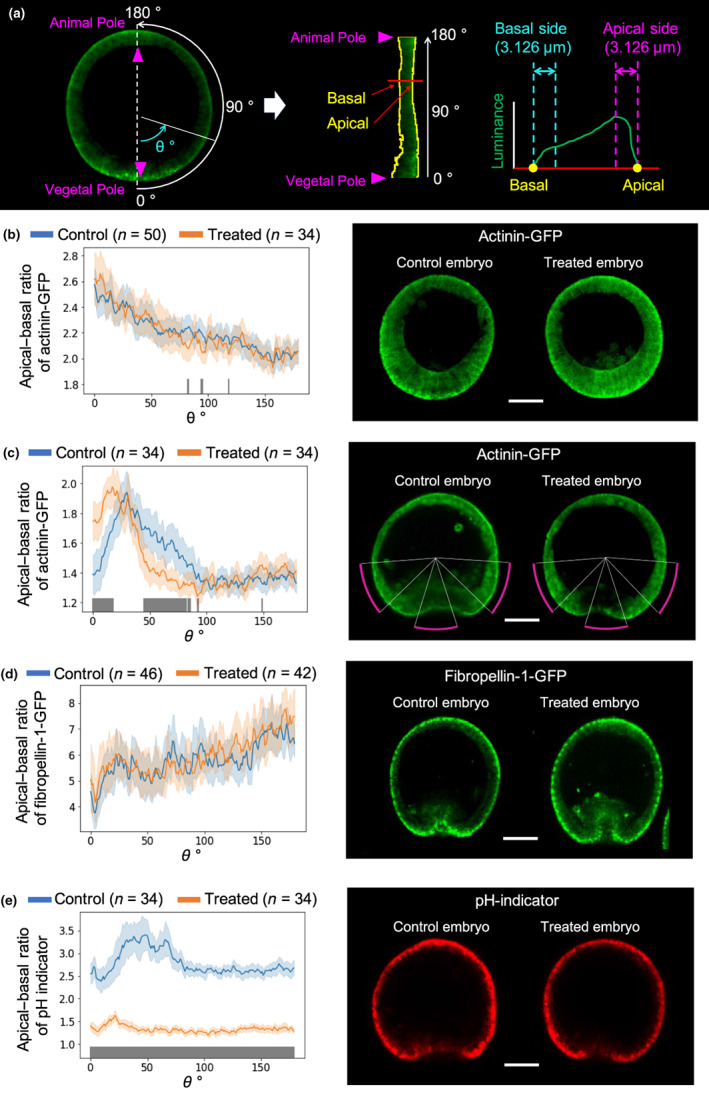
Whole embryonic distributions of actinin‐GFP, fibropellin‐1‐GFP, and pH. (a) Definitions of angle *θ* (0°–180°) from the vegetal pole (0°) to the animal pole (180°) along the circumference of the embryo cross section and apical and basal sides of cells (see also Figure S7) of confocal fluorescence microscopic images determined via actinin (actinin‐GFP: Green) intensity of embryos. (b–e) Average and 95% confidence intervals (error bars) of apical–basal ratios at angle *θ* obtained by *n* samples (left), and typical confocal fluorescence microscopic images (scale bars: 30 μm) (right) of actinin‐GFP intensities at step 1 (b) and step 2 (c), fibropellin‐1‐GFP intensities at step 2 (d), and pH indicator intensities at step 2 (e). In the left‐hand side images, blue and orange curves and bars represent the control and treated embryo values, respectively, where gray bars indicate significantly different average values between the control and treated embryos according to Welch's *t*‐test (*p* < .05) (see also Figure S1). Magenta curves in the right panel of (c) were included along the region with significant differences between the values of the control and treated embryos. The correlation coefficients of apical–basal ratios between actinin‐GFP intensities (c) and pH indicator intensities (e) in control and treated embryos were 0.56 and 0.64, respectively

The intracellular distribution of F‐actin was analyzed at the primary invagination stage (steps 1 and 2) to reveal the mechanism underlying the morphological changes in the treated embryos at the secondary invagination stage (step 4). F‐actin was visualized via fluorescence imaging using actinin‐green fluorescent protein (GFP) fusion protein where actinin was known as one of the F‐actin binding proteins (Edlund et al., 2001). The apical–basal polarity of intracellular F‐actin distribution was evaluated by estimating the fluorescence intensity ratio between the apical and basal sides of cells (Figure 3a).

Similar apical–basal ratio distributions of the actinin‐GFP signal were obtained between the control and treated embryos, while the ratios gradually increased closer to the vegetal pole in both embryos at step 1 (Figures 3b, S1a, and S2a–d). The distribution of the apical–basal ratios of actinin‐GFP signal intensities in step 2 was similar between control and treated embryos only in the animal half (Figures 3c, S1b, and S2e–h). However, from the equator to the vegetal pole, the ratio in the control embryos was larger than that in the treated embryos, whereas at the vegetal pole and its surrounding region, control embryos showed a smaller ratio than the treated embryos. Therefore, the formation of an anomalous embryonic shape of treated embryos could correlate with the deviation of apical–basal polarity of F‐actin distribution in the vegetal half of the embryos during primary invagination stage. Meanwhile, omeprazole did not influence the whole embryonic distribution of fibropellin‐1 (Figures 3d and S1c), an F‐actin scaffold in the apical lamina of the apical pole in each cell (Burke et al., 1998; Nakajima & Burke, 1996).

Since omeprazole is a proton pump inhibitor, intracellular pH was estimated using a pH indicator; fluorescent intensity increased as pH decreased. In both control and treated embryos in step 2, the apical–basal ratio of the pH indicator was greater than 1 (Figures 3e, S1d, and S3), indicating that the pH of the apical side of each cell was always lower than that on the basal side. Additionally, this ratio was higher in the vegetal half than that in the animal half of the control embryo, while this pattern disappeared in the treated embryo. Thus, the apical–basal polarity of the pH indicator fluorescent intensity was positively correlated with that of actinin‐GFP signal intensity in whole embryos (Figure 3c,e).

### 2.3 Gastrulation starts but does not progress as normal in cytoskeleton regulator‐knockout embryos

**FIGURE 4:**
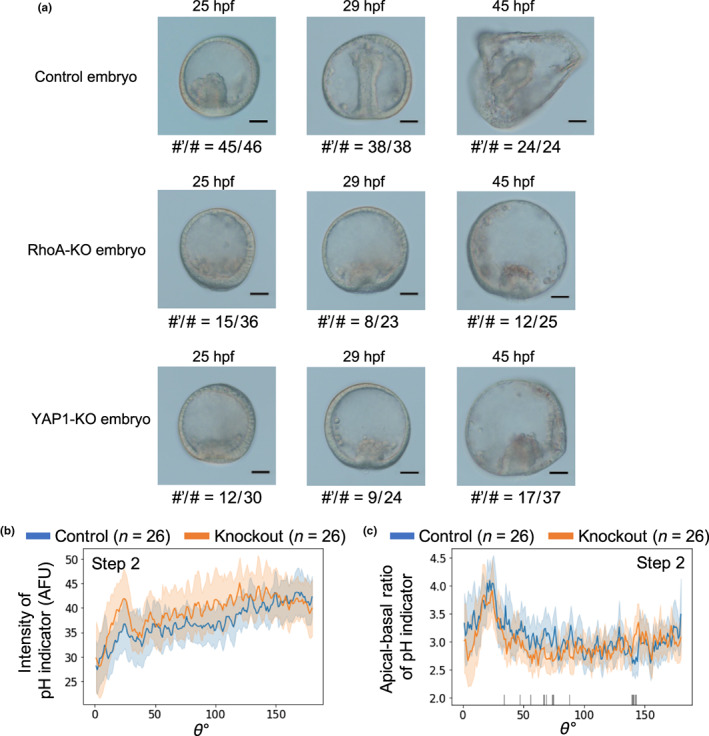
Effect of F‐actin regulator‐knockout on gastrulation. (a) Bright‐field images of gastrulation in control, *RhoA*‐knockout, and *YAP1*‐knockout embryos at selected time‐points. Primary mesenchyme cells and pigment cells were observed in all embryos suggesting that development did not stop. The knockout embryos did not form the structure like prism larva observed at 45 hpf in the control embryo (scale bars: 30 μm). # and #’ refer to the total number of sampled embryos and the number of embryos with similar shape to the image, respectively. (b and c) Average fluorescence intensities (arbitrary fluorescence units) and 95% confidence intervals (error bars) of intracellular pH indicator (b) and apical–basal ratio of pH indicator (c) of *RhoA*‐knockout embryos and control embryos as a function of angle *θ*. The indications of colors and *θ* are stated in Figures 3 and S1

CRISPR‐Cas9‐mediated knockout of the typical enhancer and repressor of F‐actin formation, *RhoA* and *YAP1* (Beane et al., 2006; Dupont et al., 2011), was performed by microinjection of *Cas9* mRNA and sgRNAs designed (Figure S4) to elucidate the contribution of F‐actin to gastrulation. Although the mutation frequencies of *RhoA* and *YAP1* knockouts were 55.6% and 77.8%, respectively, and the frameshift rate was 22.3% in either knockout (Figure S4), gastrulation stopped at step 2 at the primary invagination stage in *RhoA* or *YAP1* knockout embryos (Figure 4a); further extension of the archenteron was not observed in both knockout embryos. Interestingly, the phenotypes between these knockout embryos were similar, although the gene functions were different (Figure S4). Pigmented cells were observed at 45 hpf, indicating that development had not ceased. This showed that primary invagination occurred even in embryos with mild perturbation of F‐actin function, but subsequent gastrulation processes required appropriate F‐actin‐derived forces.

The whole embryonic distribution of average intensity and apical–basal ratios of the pH indicator were unchanged in *RhoA*‐knockout embryos compared with those in the control, and therefore, perturbation of F‐actin polymerization did not affect the pH gradient of the cell (Figures 4b,c and S1e,f).

### 2.4 Mathematical model of embryonic shape formations during early gastrulation considering cell‐dependent apical–basal intracellular polarity of F‐actin distribution

**FIGURE 5:**
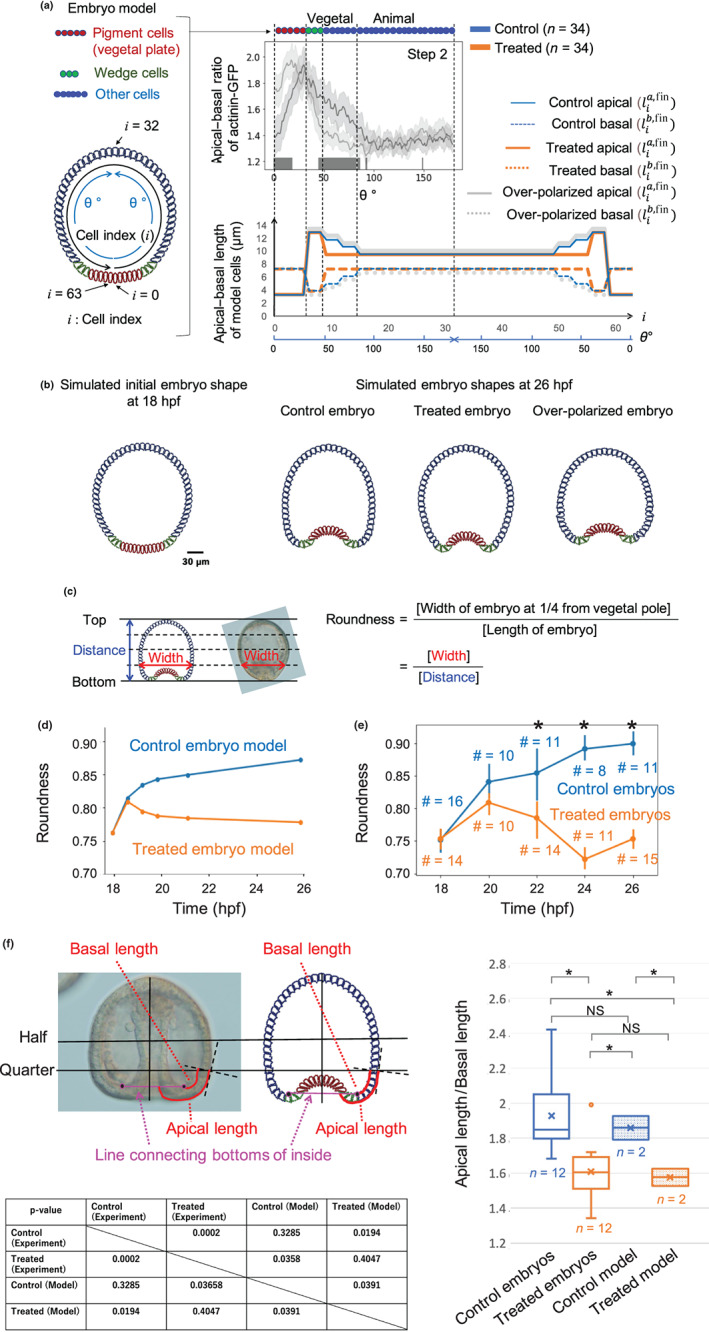
Coarse‐grained model and simulation of control, treated, and over‐polarized embryos. (a) Modeled cell lengths of apical and basal sides of control, treated, and over‐polarized embryos; $l_{i}^{a,\mathrm{fin}}$ and $l_{i}^{b,\mathrm{fin}}$ refer to the final length of the apical and basal sides of the *i*th cell, respectively (see Experimental procedures). Distributions of the final length of the former two models were determined based on the distributions of apical–basal ratios of actinin‐GFP intensities from control and treated embryos in step 2. The top center panel was a modification of Figure 3d. Red, green, and blue circles in the top panel and panel (b) represent pigment cells, wedge cells, and other cells, respectively. (b) Snapshots of the initial embryo shape at 18 and 26 hpf of the three models. (c) Definition of the roundness of the vegetal side of embryos from simulation results and imaging. (d and e) Roundness indices of vegetal sides of modeled control and treated embryos (d) and experimentally determined values (e). Blue and orange # refer to the sampling number of control and treated embryos at each time point, error bars indicated 95% confidence intervals, and * indicated that the roundness of control embryos was significantly larger than that of treated embryos according to Welch's *t*‐test (*p* < .05, see Figure S5b). (f) The ratio of apical/basal lengths obtained by experimental and simulation data, and *p*‐values of Welch's *t*‐test between the two types of data, where * and NS between two box plots indicate *p* < .05 and *p* > .05, respectively. Embryos at step 4 (late gastrula) and simulation final step were compared

A mathematical model of cell motion at the cross section including animal and vegetal poles during steps 1–4 of gastrulation was constructed to examine the influence of intracellular F‐actin distribution on the formation of embryonic shapes. The model was constructed based on the gastrulation model of *Nematostella vectensis* (Tamulonis et al., 2011) that consisted of cells constructed using springs and beads circularly connected to form a two‐dimensional cross section of the embryo. The following assumptions were made based on experimentally known facts:

1. Each embryo contained three cell types as follows: pigment cells (Kimberly & Hardin, 1998; Kominami & Takata, 2004), wedge cells (Burke et al., 1991), and other cells (Figure 5). The pigment cells near the cavity entrance were considered to be bottle‐shaped (Kimberly & Hardin, 1998; Kominami & Takata, 2004) because of the site‐specific force in these cells. This may explain the primary invagination occurring in a surgically isolated vegetal plate (Ettensohn, 1984). In the knockout experiment shown in Figure 4, partial F‐actin function may be enough for the formation of bottle‐shaped pigment cells and subsequent primary invagination. In the present model, such effect was represented by the fact that the apical side of the pigment cell was assumed smaller than those of other cells, and the apical side of each wedge cell was assumed larger than the basal side (Figure 5a). The apical and basal sizes of cells in the initial state (Figure 5b) were determined by those with which the shape of the model after the relaxation of interaction energy could imitate the embryo shape in step 1 (Figure 1).
2. The width of the apical and basal sides changed in a cell‐dependent manner in control and treated embryos due to cytoskeletal forces generated by F‐actin. In the present model, the F‐actin concentration in each side of the cell was assumed to correlate positively with the cell cortical force generations that push out each cell side area to stretch tissues due to F‐actin polymerization (Footer et al., 2007; Mullins & Hansen, 2013; Pollard et al., 2000; Pollard & Cooper, 2009). Thus, the lengths of the apical and basal sides of the cells, except pigment cells and wedge cells, were determined by referring the profile of the apical–basal ratio of actinin‐GFP in step 2 (Figure 5a). Here, the elongations of the apical side of these cells were determined based on the apical–basal ratio of actinin‐GFP, and the length of the basal side was assumed to change oppositely to the apical side to maintain the cell area.
3. Cell divisions and three‐dimensional mutual cell invasions mainly contributing to late gastrulation were not included because these processes were rarely observed in early gastrulation (Mizoguchi, 1999). Therefore, the present model described embryo shape dynamics of the control and treated embryos introducing two‐dimensional motions and deformations of the apical and basal sides in 64 cells (Figure 5a).

Simulations showed different shapes (Figure 5b), although each shape was similar to that experimentally observed at step 4 (Figure 1). Similar temporal changes in roundness indices (Figure 5c) of control embryos (0.85–0.9) and treated embryos (0.75–0.8) were observed in the simulated (Figures 5d and S5a) and experimental data (Figures 5e and S5b). Additionally, the ratio of apical/basal lengths along the embryos around the vegetal pole obtained at the final time step in the simulations of both control and the treated model exhibited similar features to those of the control and treated embryos at step 4 (late gastrula), respectively (Figure 5f). Here, the control embryos and control model showed significantly larger ratios than the treated embryos and treated model, respectively, but there were no significant differences between the observed embryos and simulation results in both the control and treated conditions.

The simulation where the elongation ratio of the apical side of each cell was a little larger than that of control was also performed, named “over‐polarized embryo” (Figure 5a,b). The whole embryonic shape of this model showed in a wider and larger roundness index than the control embryos (Figure S5a). This suggested that the site‐specific apical–basal ratio polarity of F‐actin distribution in each cell influenced sensitively the whole embryonic shape.

## 3 DISCUSSION

Omeprazole treatment resulted in abnormal embryo shape with incomplete gastrulation, in particular, more than half of treated embryos showed partial exogastrulation with outward extending endodermal tissue (Figures 1 and 2). Such anomalous morphogenesis was due to the perturbation of intracellular F‐actin distribution in the vegetal half of embryos (Figures 3 and 5) as follows.

F‐actin concentrations generating cell cortical forces were significantly larger at the apical side of each cell than that of the basal side in the vegetal half of the control embryos, except for cells at the vegetal pole. Therefore, the apical planes of wedge cells and other cells in the vegetal pole side were expected to be significantly wider than the basal planes. Conversely, the apical planes of cells in the vegetal half of the treated embryo were not significantly wider compared to the basal sides due to lower apical F‐actin levels. Simulation results showed that such expected features in the vegetal half may lead to normal early gastrulation in control embryos and partial exogastrulation in treated embryos (Figure 5). Therefore, appropriate embryonic intracellular apical F‐actin distribution in the vegetal half is required for gastrulation.

Although the present mathematical model assumed that F‐actin concentration correlates positively with the forces elongating each cell periphery to stretch tissues, the force by F‐actin varies both qualitatively and quantitatively depending on its interaction with the environment and other molecules. At least for the morphogenetic process considered in this study, the employed assumptions for the relationship between F‐actin concentration and the elongation of each side of the cells were supported by the relationships between the apical and basal lengths in the control and treated embryo after morphological changes (Figure 5f).

Gastrulation does not proceed when apical lamina is inhibited (Burke et al., 1991). The whole embryonic distributions of apical lamina along cells were nearly identical between the control and treated embryos, although large differences were observed between the cell shapes in the vegetal half and whole embryos. This indicated that the generated force was independent of the relative amounts of the apical lamina in each cell. Embryos with a partial knockout of F‐actin‐regulating factors (*RhoA* or *YAP1*) showed inhibited gastrulation at the primary invagination step (Figure 4a,b). This indicated that the secondary invagination was inhibited without appropriately regulated F‐actin, even if the apical lamina existed. Therefore, the apical lamina may play the role of just the scaffold for apical F‐actin, and F‐actin generates the force required for deformation processes driving the secondary invagination of gastrulation and whole embryonic deformation. Notably, the importance of the role of intracellular F‐actin for gastrulation was also supported by the recent study performing Rab35 knockdown (Remsburg et al., 2021).

The elongation of the archenteron requires cell divisions and rearrangements (Ettensohn, 1985). Conversely, to focus on the morphogenetical process independent of cell divisions or those during early gastrula stage with little occurrence of cell divisions, the present mathematical model did not involve the effects of cell divisions. Therefore, the model could not reproduce cell division‐induced morphological processes like elongations of archenteron of normal embryos and more outward protruding of the part of the elongated archenteron in treated embryos (Figure 1). However, the morphological features evaluated by the temporal change in the roundness index and the lengths of apical and basal sides of the vegetal side of both control and treated embryos, which should be spatially and temporally little influenced by cell divisions, were well reproduced in our models (Figure 5d–f). These facts suggested that not only cell divisions but also the cytoskeletal forces provide dominant contributions to gastrulation. The considerations of the model implementing the effects of cell divisions are important and are considered as a future issue for not only the simulations of whole gastrulation processes but also the considerations of the regulatory mechanism of post gastrulation processes like left–right asymmetry formations of embryos as mentioned previously.

The present mathematical models were constructed based on the results of the apical–basal ratios of actinin‐GFP of step 2 to simulate the morphological features of step 3 and beyond (Figure 5a). It was noted that higher values of the apical–basal ratio of actinin‐GFP intensities were obtained at step 1 than at step 2 (Figure 3b,c). This may be explained by the fact that the cells at step 1 are larger than those at step 2 (Figure 3b,c), because the apical–basal difference between the molecule concentrations was expected to increase with the increases in apical–basal distance. Additionally, the absolute values of actinin‐GFP intensities in the apical and basal sides of cells in both control and treated embryos at step 1 were sufficiently smaller than those at step 2 (Figure S2). These findings suggest that the contributions of the apical–basal ratios of actinin‐GFP intensities at step 2 were more dominant than those at step 1 for the focused gastrulation processes.

CRISPR‐Cas9‐mediated *RhoA*‐knockout showed an intermediate level (55.6%) of mutation frequency (Figure S4) and the knockout embryos exhibited primary invagination. However, *Lytechinus variegatus* (LV) embryos expressing dominant‐negative *RhoA* failed to initiate primary invagination (Beane et al., 2006). Therefore, the contribution of *RhoA* is expected to be considerable also for the primary invagination, but the extent of the dependency on F‐actin function is small and a mechanism other than the F‐actin‐related system might be involved in this initial process, at least in HP embryos. F‐actin function may be essential for secondary invagination.

F‐actin network formation, generating cell cortical forces, was drastically enhanced with decreased pH in vitro (Köhler et al., 2012). Whole embryonic distributions of apical–basal ratios of the pH indicator at step 2 were found to correlate positively with those of intracellular actinin‐GFP signal intensities (Figure 3c,e), suggesting that F‐actin concentration was high when the pH was low at the apical side of cells. Additionally, the apical–basal polarity of F‐actin distribution in the vegetal half cells of the omeprazole‐treated embryo decreased to significantly smaller than those of control embryo (Figure 3c) while whole embryonic distribution of the apical lamina did not exhibit any significant changes (Figure 3d). Conversely, the whole embryonic distribution of intracellular pH and polarity was unchanged even when F‐actin polymerization was directly perturbed by the knockout of F‐actin‐regulating factors (Figure 4d). These facts suggested that the intracellular pH is a one‐sided regulator of F‐actin polymerization also in HP embryos and contributes to the progression of the secondary invagination to form appropriate apical–basal intracellular polarity of F‐actin distribution.

Herein, omeprazole increased the intracellular pH in most cells in the HP embryo (Figure S6) conversely from the apical–basal polarities of intracellular pH. Oppositely from the case of HP embryo, a recent study of the LV embryo reported that omeprazole significantly decreases intracellular pH (Schatzberg et al., 2015). This suggests that the apical–basal polarities of intracellular pH in LV embryo were increased by omeprazole. The influence of pH on F‐actin network formation and force generation might be a universal biochemical feature as confirmed via both in vitro experiments (Köhler et al., 2012) and the present in vivo study. Therefore, omeprazole may increase the apical–basal polarities of intracellular pH and F‐actin concentration in LV embryo similar to the “over‐polarized embryo” model. The over‐polarized embryo model exhibited a wider embryonic shape with a larger roundness index than the control embryos (Figures 5b and S5b), which is consistent with LV imaging (Schatzberg et al., 2015). This also supported the significant contributions of cell position‐dependent intracellular F‐actin apical–basal polarizations to early gastrulation.

Omeprazole treatment of sea urchin embryos disrupts the left–right asymmetric formations of the adult rudiment although gastrulation is completed (Bessodes et al., 2012; Duboc et al., 2005; Hibino et al., 2006) (Figure S7). The left–right asymmetric *nodal* gene expression observed immediately after gastrulation is important for establishing this asymmetry (Duboc et al., 2005). Disrupted left–right asymmetry in HP is observed at lower (60%–80%) omeprazole concentrations (Hibino et al., 2006), different to what was observed in the present study. Dilute omeprazole solutions induced weak perturbation of gut formation in the present study, which may disrupt cell–cell interactions and regulate whole embryonic *nodal* gene expression.

This report revealed the cell position‐dependent regulations of intracellular F‐actin polymerization and polarization by intracellular pH by focusing on the normal and omeprazole‐induced abnormal gastrulation in sea urchins. Frogs, chickens, zebrafish, and ascidians disrupted left–right asymmetry upon inhibition of H^+^/K^+^ ion pump activity (Kawakami et al., 2005; Levin et al., 2002; Shimeld & Levin, 2006). Therefore, omeprazole may disrupt left–right asymmetry of sea urchin embryo through the inhibition of H^+^/K^+^ ion pump activity (Bessodes et al., 2012; Duboc et al., 2005; Hibino et al., 2006), which suggests the involvement of H^+^/K^+^ ion pump in intracellular pH distribution regulating F‐actin distribution. Due to the high similarity between H^+^/K^+^ ion pump and Na^+^/K^+^ ion pump sequences in sea urchin, however, the detail features of H^+^/K^+^ ion pump such as whole embryonic gene expression profiles along the developmental process and intracellular distributions have yet to be determined; hence, further investigations are warranted to explore the formation mechanism underlying embryonic position‐dependent intracellular states promoting gastrulation. The universal effect of inhibiting H^+^/K^+^ ion pump activity on early embryo formation among different animals should also be determined in the future.

## 4 EXPERIMENTAL PROCEDURES

### 4.1 Animals and embryos

Adult Japanese sea urchins (HP) were collected from the Seto Inland Sea or Tateyama Bay. Eggs and sperms were obtained via coelomic injection of 0.55‐M KCl. Fertilized eggs were cultured in filtered seawater at 16°C.

### 4.2 Omeprazole treatment

A 100‐mM stock solution of omeprazole (FUJIFILM Wako Pure Chemical Corporation) dissolved in dimethyl sulfoxide was immediately added to fertilized eggs at a final concentration of 100 μM and cultured at 16°C.

### 4.3 Whole mount in situ hybridization

Whole mount in situ hybridization of glial cells missing (*gcm*) was performed following the method of Minokawa et al. (2004). Antisense RNA probe of *gcm* was transcribed from a PCR fragment amplified from the cDNA clone with those primers stated in Table S1. Antisense RNA probe was synthesized using MEGAscript T7 Transcription Kit (Ambion) with DIG RNA Labeling Mix (Roche).

Sea urchin embryos were fixed by fixative III [4% paraformaldehyde, 32.5% filtered seawater, 32.5‐mM 3‐(*N*‐morpholino) propane sulfonic acid (MOPS) (pH 7.0), and 162.5‐mM NaCl] for 16 h at 4°C. Fixed embryos were preserved in 70% ethanol at −20°C until use. Embryos were washed three times with 100‐mM MOPS, 500‐mM NaCl, and 0.1% Tween20 (MOPS buffer). The embryos were prehybridized for 3 h in the hybridization buffer containing 70% formamide, 100‐mM MOPS (pH 7.0), 500‐mM NaCl, 0.1% Tween20, and 1 mg/ml of bovine serum albumin (BSA) at 50°C. For hybridization, 0.1 ng/μl of probe was added to the prehybridized samples, and the hybridization was performed at 50°C with shaking for 6 days. After hybridization, the embryos were washed five times with MOPS buffer. The embryos were then transferred to probe‐free hybridization buffer again at 50°C for 3 h and washed three times with MOPS buffer. For blocking, the embryos were incubated in MOPS buffer containing 10 mg/ml of BSA for 20 min at room temperature (RT), followed by additional incubation in the MOPS buffer containing 10% sheep serum and 1 mg/ml of BSA for 30 min at 37°C. Incubation with a 1:1500 dilution of the alkaline phosphatase conjugated Fab fragments (Roche Molecular Biochemicals) in the MOPS buffer containing 1% sheep serum and 1 mg/ml of BSA was performed overnight at 4°C. Embryos were washed five times every 2 h to remove antibodies with MOPS buffer (the last wash was done with overnight shaking). The embryos were washed two times with alkaline phosphatase buffer [100‐mM Tris HCl (pH 9.5), 50‐mM MgCl_2_, 100‐mM NaCl, and 1‐mM Levamisole]. The staining reaction was done in the alkaline phosphatase buffer containing 10% dimethylformamide and nitro blue tetrazolium (NBT) (SIGMA) and 5‐bromo‐4‐chloro‐3‐indolyl‐phosphate (BCIP) (SIGMA).

### 4.4 Alkaline phosphatase staining

Alkaline phosphatase staining of endodermal tissue was performed following Whittaker and Meedel (1989) with modification. Sea urchin embryos were fixed by fixative III (4% paraformaldehyde, 32.5% filtered seawater, 32.5‐mM MOPS, pH 7.0, and 162.5‐mM NaCl) for 1 h at 4°C. Embryos were washed three times using 0.5‐ml 1× phosphate‐buffered saline. Embryos were reacted in reaction buffer [100‐mM Tris–HCl (pH 9.5), 100‐mM NaCl, and 5‐mM MgCl_2_] containing NBT (SIGMA) and BCIP (SIGMA). Embryos showed specific staining when reacted at 4°C for 48 h and then at RT for 6 h.

### 4.5 Live imaging of pH, actinin, and apical lamina

Intracellular pH of sea urchin embryos was visualized using pHrodo Red AM intracellular pH indicator or 5‐(and‐6)‐carboxy SNARF‐1 (C‐1270) (Thermo Fisher Scientific) at final concentrations of 10 and 5 μM, respectively, and stained at 16°C for 30 min. The embryos were washed in filtered seawater and observed using confocal microscopy with laser illumination at 555/585 nm excitation/emission for pHrodo and 555/573 m excitation/emission for SNARF‐1, respectively.

Actinin and fibropellin‐1 were visualized following the fusion of the proteins with GFP. RNA was extracted from mesenchyme blastula stage HP embryos using ISOGEN (Nippongene) according to the manufacturer's instructions. Actinin‐ and fibropellin‐1‐coding sequences were amplified via reverse transcription‐polymerase chain reaction (RT‐PCR) using SuperScript™ III Reverse Transcriptase (Thermo Fisher Scientific) with the primers stated in Table S2 and cloned into pGreenLantern2‐derived plasmid at EcoRI and XhoI restriction sites. mRNA (actinin‐GFP, fibropellin‐1‐GFP, in that order) was synthesized in vitro using an mMESSAGE mMACHINE T7 ultra transcription kit (Thermo Fisher Scientific) and purified using an RNeasy mini kit (Qiagen). mRNA was microinjected into fertilized eggs as described by Liu et al. (2019).

Fluorescent images were acquired by confocal microscopy using an excitation of 488 nm and emission of 515 nm. The images of the animal and vegetal poles of the embryo were analyzed based on the *z*‐stacked image with the largest area by averaging seven *z*‐axis images at a total thickness of 6 μm.

### 4.6 Quantification of fluorescent signals

Fluorescent images of embryos were acquired according to the angle *θ* (0°–180°) between the vegetal and animal poles along the circumference (Figures 3a and S7) and transformed into a band‐like image using the “Polar Transformer” function (https://imagej.nih.gov/ij/plugins/polar-transformer.html) of ImageJ 2.1.0. Each band‐like image was filtered using a median filter (radius = 1.0). The filtered images were binarized using “Mean” (pHrodo indicator), “IsoData” (actinin), and “Triangle” (fibropellin‐1 and SNARF‐1) functions, respectively, to obtain the cellular regions of the embryo. The apical–basal ratios of the pH indicator, actin‐GFP, and fibropellin‐1‐GFP for *θ* were determined using average fluorescent intensities over the regions at a width of 3.126 μm from the apical and basal sides (Figures 3a and S7).

The fluorescent intensity of the pHrodo indicator was defined as the average fluorescent intensity over the intracellular region at a width of 6.232 μm from the apical side (Figure S7). Fluorescent signal intensity values were estimated by calculating the ratio between the observed fluorescence intensity and the average background fluorescence intensity around the entire embryo.

F‐actin and apical lamina were stained via mRNA microinjections of actinin‐GFP and fibropellin‐1‐GFP into fertilized eggs. Nonnegligible variations was inevitable in the concentration of injected mRNA among fertilized egg samples, which would be amplified during development. Therefore, only the apical–basal ratios were mainly used to evaluate the intracellular features of F‐actin and apical lamina distributions. For the fluorescence intensity of actinin‐GFP in the apical and basal sides of cells (see Figure S2).

Two data points were obtained for each *θ* from each fluorescence image of the embryo since the left and right sides of the embryo were considered axis symmetric against the animal–vegetal axis. Both data were used to estimate the sample average and 95% confidence interval of each value, where the number of samples (*n*) was given as 2 × (number of observed embryos).

### 4.7 Gene knockout using CRISPR‐Cas9

Knock out of endogenous *RhoA* and *YAP1* in HP was performed using the method described by Liu et al. (2019). The oligonucleotide sequences used for sgRNA preparations are listed in Table S3.

### 4.8 Heteroduplex mobility assay and DNA sequencing

Analysis of knockout embryos using heteroduplex mobility assay and DNA sequencing was performed as previously published by Liu et al. (2019). The primer sequences used to amplify each target region are listed in Table S4.

### 4.9 Mathematical model of sea urchin embryos

A two‐dimensional particle model describing cellular motion at the cross section of animal poles and vegetal poles of sea urchin embryos during steps 1–3 of gastrulation was constructed. The determined number of cells was based on that at the equatorial plane of the embryo at the blastula stage (Mizoguchi, 1999). The following assumptions were made: (1) each cell was represented by 16 particles with radii (*r*) of 1.125 μm, and each embryo was represented by 64 cells connected in a ring (Figure S8); (2) each cell perimeter was 36 μm and the height and width of the embryo at step 1 were 110 and 100 μm, respectively, which was consistent with sea urchin embryo observations (results are not shown); (3) the number of cells was constant because cell divisions and cell invasions from other cross sections were rarely observed during the primary invagination stage (Mizoguchi, 1999); (4) the motion of each particle obeyed the overdamped limit of the Equation (1) of motion,

(1) $$\frac{\partial X_{i,j}}{\partial t}=-\frac{1}{\gamma }\frac{\partial V}{\partial X_{i,j}},$$

where $X_{i,j}=\left(x_{i,j}\left(t\right),y_{i,j}\left(t\right)\right)$ is the position of the *j*th particle constructing the *i*th cell (*i* = 0, 1, 2, … 63 and *j* = 0, 1, 2, … 15) on the *x*–*y* plane at time *t* (Figures 5 and S8), *γ* is the coefficient of the drag force acting on each particle, and *V* is the potential of the entire system; and (5) the *y*‐axis was parallel to the animal–vegetal axis of the embryo model.

We rewrote $\frac{V}{\gamma }=U$ and U was calculated as follows:

(2) $$U=U_{\text{bond}}+U_{\text{collision}}+U_{\text{actin}}+U_{\text{adhesion}}+U_{\mathrm{ca}}+U_{\mathrm{ea}},$$

where $U_{\text{bond}}$ is the elastic force potential between each neighboring pair of particles in each cell to maintain each cell perimeter, denoted as follows:

(3) $$U_{\text{bond}}=\sum_{i=0}^{63}\sum_{j=0}^{15}\frac{k_{b}}{2}{\left(d_{\left(i,j\right),\left(i,j+1\right)}-2r\;\right)}^{2},$$

where $k_{b}$, $d_{\left(i,j\right),\left(i,j+1\right)}$, and *r* are the coefficient of elasticity, the distance between the *j*th and *j* + 1th particle (j+1=0 for j=15) in the *i*th cell at time *t*, and the particle radius, respectively.

$U_{\text{collision}}$ is the potential of excluded volume effects among all particles denoted as follows:

(4) $$U_{\text{collision}}=\sum_{i<k}\sum_{j<l}\theta \left(2r-d_{\left(i,j\right),\left(k,l\right)}\right)\frac{k_{c}}{2}{\left(2r-d_{\left(i,j\right),\left(k,l\right)}\right)}^{2},$$

where $k_{c}$ is the coefficient of repulsion between two particles, and $\theta \left(x\right)$ is the Heaviside step function.

$U_{\text{actin}}$ is the elastic force potential to form and sustain cell shape with the expansion and contraction of the apical and basal sides by the cytoskeleton, denoted as follows:

(5) $$U_{\text{actin}}=\sum_{i}\frac{k_{a}}{2}\left({\left(d_{\left(i,10\right),\left(i,14\right)}-l_{i}^{a}\;\right)}^{2}+{\left(d_{\left(i,2\right),\left(i,6\right)}-l_{i}^{b}\;\right)}^{2}\right),$$

where the apical and basal sides of the *i*th cell consisted of particles with *j* = 10–14 and *j* = 2–6, respectively. The wideness of the apical and basal sides at time *t* was given by $d_{\left(i,10\right),\left(i,14\right)}$ and $d_{\left(i,2\right),\left(i,6\right)}$, respectively. The basic wideness of the apical and basal sides was given by $l_{i}^{a}$ and $l_{i}^{b}$, respectively. $k_{a}$ was assumed by the coefficient of elasticity to sustain the wideness of the apical and basal sides of the cell.

$U_{\text{adhesion}}$ is the potential force for cell adhesion by proteins, such as cadherin, denoted as follows:

(6) $$U_{\text{adhesion}}=\sum_{i}\sum_{j}\frac{k_{h}}{2}{\left(d_{\left(i,j\right),\left(i+1,k\left(j\right)\right)}-2r\;\right)}^{2},$$

where $k_{h}$ is the coefficient for the adhesive forces between the particles of the *i*th and *i* + 1th cells (i+1=0 for i=63), and $k\left(j\right)\equiv 6,7,8,9,10$ for j≡2,1,0,15,14, respectively (Figure S8).

$U_{\mathrm{ca}}$ is the potential of the forces to conserve each cell area, denoted as follows:

(7) $$U_{\mathrm{ca}}=\sum_{i}\frac{k_{\mathrm{ca}}}{2}{\left(s_{i}-s_{i}^{\mathrm{ini}}\right)}^{2},$$

where $k_{\mathrm{ca}}$ is the coefficient of elasticity required to maintain each cell area. The area of the *i*th cell was estimated by $s_{i}=\sum_{j=0}^{15}\left|X_{i,j}-X_{i}^{\mathrm{cc}}\right|$ with $X_{i}^{\mathrm{cc}}=\frac{1}{16}\sum_{j=0}^{15}X_{i,j}$, and $s_{i}^{\mathrm{ini}}$ referred to $s_{i}$ given by $X_{i,j}$ at the initial state.

$U_{\mathrm{ea}}$ is the potential of the forces to maintain the area (volume) of the sea urchin embryo, denoted as follows:

(8) $$U_{\mathrm{ea}}=\frac{k_{\mathrm{ea}}}{2}{\left(S-S^{\mathrm{ini}}\right)}^{2},$$

where $k_{\mathrm{ea}}$ is the coefficient of elasticity required to maintain embryo area. The area was estimated by $S=\sum_{i=0}^{63}\left|X_{i}^{\mathrm{cc}}-X^{\mathrm{ec}}\right|$ with $X^{\mathrm{ec}}=\frac{1}{64}\sum_{i=0}^{63}X_{i}^{\mathrm{cc}}$, and $S^{\mathrm{ini}}$ referred to S given by $X_{i,j}$ at the initial state.

$U_{\mathrm{ca}}$ and $U_{\mathrm{ea}}$ were considered alternatives for the volume‐conserving forces in real 3‐D cells and embryos as internal pressures in each cell and embryo were isotropic.

### 4.10 Simulation method for the mathematical model

The simulations of the present mathematical model were performed through the integrals of the equation of motion (1) using the Euler method at time intervals of 0.000064 h with conserved $X^{\mathrm{ec}}=\left(0,0\right)$. In all models, the parameters $k_{\text{bond}},k_{\text{collision}},k_{\text{actin}},k_{\text{adhesion}},k_{\mathrm{ca}}$, and $k_{\mathrm{ea}}$ were given empirically as 9375, 6250, 8125, 8125, 625, and 0.00625 h^−1^, respectively, because there were no experiments to measure or estimate them. The qualitative features of the results were independent of the details of these values if the order was maintained. The model formed the embryo shape at step 1 of gastrulation if the appropriate cell type‐dependent values of $l_{i}^{a}$ and $l_{i}^{b}$, ${=l}_{i}^{a,\mathrm{ini}}$ and $l_{i}^{b,\mathrm{ini}}$ were given for the models of pigment cells, wedge cells, and other cells (Figure 5). This configuration gave the particle positions $X_{i,j}$ at time *t* = 0 (initial configuration) in all simulations.

The early gastrulation processes were simulated by the change in $l_{i}^{a}$ and $l_{i}^{b},$ respectively, from ${=l}_{i}^{a,\mathrm{ini}}$ and $l_{i}^{b,\mathrm{ini}}$ to ${=l}_{i}^{a,\mathrm{fin}}$ and $l_{i}^{b,\mathrm{fin}}$ at time *t* = 0. Here, $l_{i}^{a,\mathrm{fin}}$ and $l_{i}^{b,\mathrm{fin}}$ were assumed to obey $l_{i}^{a,\mathrm{fin}}+l_{i}^{b,\mathrm{fin}}=l_{i}^{a,\mathrm{ini}}+l_{i}^{b,\mathrm{ini}}=\text{constant}$ among cells except pigment cells based on the expectation that F‐actin was constant. The model showed similar structural behaviors to the early gastrulation as the relaxation process of $d_{\left(i,10\right),\left(i,14\right)}\to l_{i}^{a,\mathrm{fin}}$ and $d_{\left(i,2\right),\left(i,6\right)}\to l_{i}^{b,\mathrm{fin}}$.

### 4.11 Statistical analysis

All experiments were performed independently twice or more with four replicates or more per experiment. Statistical test was performed using SciPy library (https://www.scipy.org/).

The roundness index of the vegetal side of each embryo was evaluated using the following: (embryo width half‐way between the bottom and middle of the embryo)/(embryo length) (Figure 5c).

The apical length of embryos around vegetal pole was defined as the length of the curve along apical side from 1/4 of the height of the whole embryo to a point that this curve crosses with straight line connecting the bottom of the inner side of the embryo (Figure 5f). The basal length was defined as the curve from the bottom of the inner side of the embryo to the point at which this curve intersects the line that crosses the tangent line of the apical envelope at 1/4 of the embryo perpendicularly (Figure 5f). The same definitions were employed to determine the apical and basal lengths around vegetal pole also for simulation results (Figure 5f). Each length of the curve was measured using ImageJ 2.1.0. The number of samples (*n*) was provided as 2 × (number of observed embryos), and those from the simulation results was considered 2 since the data were obtained from the left side and the right side of each model.

## CONFLICT OF INTEREST

The authors declare that the research was conducted in the absence of any commercial or financial relationships that could be construed as a potential conflict of interest.

## AUTHOR CONTRIBUTIONS

Kaichi Watanabe, Naoaki Sakamoto, and Akinori Awazu conceived and designed the study; Kaichi Watanabe, Yuta Kurose, and Naoaki Sakamoto. conducted the experiments; Kaichi Watanabe, Yuhei Yasui, Masashi Fujii, and Akinori Awazu analyzed the data; Kaichi Watanabe and Akinori Awazu conducted the mathematical model construction and simulations; Kaichi Watanabe, Naoaki Sakamoto, and Akinori Awazu wrote the manuscript with support from all authors; and Takashi Yamamoto supervised the work.

## Supporting information


**Appendix S1** Supporting Information

## References

[gtc12934-bib-0001] Aihara, M. , & Amemiya, S. (2001). Left–right positioning of the adult rudiment in sea urchin larvae is directed by the right side. Development, 128(24), 4935–4948. 10.1242/dev.128.24.4935 11748131

[gtc12934-bib-0002] Beane, W. S. , Gross, J. M. , & McClay, D. R. (2006). RhoA regulates initiation of invagination, but not convergent extension, during sea urchin gastrulation. Developmental Biology, 292(1), 213–225. 10.1016/j.ydbio.2005.12.031 16458878

[gtc12934-bib-0003] Bessodes, N. , Haillot, E. , Duboc, V. , Röttinger, E. , Lahaye, F. , & Lepage, T. (2012). Reciprocal signaling between the ectoderm and a mesendodermal left–right organizer directs left–right determination in the sea urchin embryo. PLoS Genetics, 8(12), e1003121. 10.1371/journal.pgen.1003121 23271979 PMC3521660

[gtc12934-bib-0004] Burke, R. D. , Lail, M. , & Nakajima, Y. (1998). The apical lamina and its role in cell adhesion in sea urchin embryos. Cell Adhesion and Communication, 5(2), 97–108. 10.3109/15419069809040284 9638331

[gtc12934-bib-0005] Burke, R. D. , Moller, D. J. , Krupke, O. A. , & Taylor, V. J. (2014). Sea urchin neural development and the metazoan paradigm of neurogenesis. Genesis, 52(3), 208–221. 10.1002/dvg.22750 25368883

[gtc12934-bib-0006] Burke, R. D. , Myers, R. L. , Sexton, T. L. , & Jackson, C. (1991). Cell movements during the initial phase of gastrulation in the sea urchin embryo. Developmental Biology, 146(2), 542–557. 10.1016/0012-1606(91)90255-2 1864470

[gtc12934-bib-0007] Dan, K. , & Okazaki, K. (1956). Cyto‐embryological studies of sea urchins. III. Role of the secondary mesenchyme cells in the formation of the primitive gut in sea urchin larvae. The Biological Bulletin, 110(1), 29–42. 10.2307/1538890

[gtc12934-bib-0008] Davidson, E. H. , Rast, J. P. , Oliveri, P. , Ransick, A. , Calestani, C. , Yuh, C. H. , Minokawa, T. , Amore, G. , Hinman, V. , Arenas‐Mena, C. , Otim, O. , Brown, C. T. , Livi, C. B. , Lee, P. Y. , Revilla, R. , Schilstra, M. J. , Clarke, P. J. C. , Rust, A. G. , Pan, Z. , … Bolouri, H. (2002). A provisional regulatory gene network for specification of endomesoderm in the sea urchin embryo. Developmental Biology, 246(1), 162–190. 10.1006/dbio.2002.0635 12027441

[gtc12934-bib-0009] Davidson, L. A. , Koehl, M. A. , Keller, R. , & Oster, G. F. (1995). How do sea urchins invaginate? Using biomechanics to distinguish between mechanisms of primary invagination. Development, 121(7), 2005–2018. 10.1242/dev.121.7.2005 7635048

[gtc12934-bib-0010] Duboc, V. , Röttinger, E. , Lapraz, F. , Besnardeau, L. , & Lepage, T. (2005). Left–right asymmetry in the sea urchin embryo is regulated by nodal signaling on the right side. Developmental Cell, 9(1), 147–158. 10.1016/j.devcel.2005.05.008 15992548

[gtc12934-bib-0011] Dupont, S. , Morsut, L. , Aragona, M. , Enzo, E. , Giulitti, S. , Cordenonsi, M. , Zanconato, F. , Le Digabel, J. , Forcato, M. , Bicciato, S. , Elvassore, N. , & Piccolo, S. (2011). Role of YAP/TAZ in mechanotransduction. Nature, 474(7350), 179–183. 10.1038/nature10137 21654799

[gtc12934-bib-0012] Edlund, M. , Lotano, M. A. , & Otey, C. A. (2001). Dynamics of alpha‐actinin in focal adhesions and stress fibers visualized with alpha‐actinin‐green fluorescent protein. Cell Motility and the Cytoskeleton, 48(3), 190–200. 10.1002/1097-0169(200103)48:3<190::AID-CM1008>3.0.CO;2-C 11223950

[gtc12934-bib-0013] Ettensohn, C. A. (1984). Primary invagination of the vegetal plate during sea urchin gastrulation. American Zoologist, 24(3), 571–588. 10.1093/icb/24.3.571

[gtc12934-bib-0014] Ettensohn, C. A. (1985). Gastrulation in the sea urchin embryo is accompanied by the rearrangement of invaginating epithelial cells. Developmental Biology, 112(2), 383–390. 10.1016/0012-1606(85)90410-5 4076547

[gtc12934-bib-0015] Footer, M. J. , Kerssemakers, J. W. , Theriot, J. A. , & Dogterom, M. (2007). Direct measurement of force generation by actin filament polymerization using an optical trap. Proceedings of the National Academy of Sciences, 104(7), 2181–2186. 10.1073/pnas.0607052104 PMC1892916 17277076

[gtc12934-bib-0016] Gustafson, T. , & Kinnander, H. (1956). Microaquaria for time‐lapse cinematographic studies of morphogenesis in swimming larvae and observations on sea urchin gastrulation. Experimental Cell Research, 11(1), 36–51. 10.1016/0014-4827(56)90188-4 13356825

[gtc12934-bib-0017] Gustafson, T. , & Kinnander, H. (1960). Cellular mechanisms in morphogenesis of the sea urchin gastrula: The oral contact. Experimental Cell Research, 21(2), 361–373. 10.1016/0014-4827(60)90268-8 13709957

[gtc12934-bib-0018] Hardin, J. (1988). The role of secondary mesenchyme cells during sea urchin gastrulation studied by laser ablation. Development, 103(2), 317–324. 10.1242/dev.103.2.317 3224556

[gtc12934-bib-0019] Hardin, J. D. , & Cheng, L. Y. (1986). The mechanisms and mechanics of archenteron elongation during sea urchin gastrulation. Developmental Biology, 115(2), 490–501. 10.1016/0012-1606(86)90269-1

[gtc12934-bib-0020] Hibino, T. , Ishii, Y. , Levin, M. , & Nishino, A. (2006). Ion flow regulates left–right asymmetry in sea urchin development. Development Genes and Evolution, 216(5), 265–276. 10.1007/s00427-005-0051-6 16534626

[gtc12934-bib-0021] Hoshi, M. (1979). Exogastrulation induced by heavy water in sea urchin larvae. Cell Differentiation, 8(6), 431–436. 10.1016/0045-6039(79)90039-3 299708

[gtc12934-bib-0022] Kawakami, Y. , Raya, A. , Raya, R. M. , Rodríguez‐Esteban, C. , & Belmonte, J. C. I. (2005). Retinoic acid signalling links left–right asymmetric patterning and bilaterally symmetric somitogenesis in the zebrafish embryo. Nature, 435(7039), 165–171. 10.1038/nature03512 15889082

[gtc12934-bib-0023] Khurrum, M. , Hernandez, A. , Eskalaei, M. , Badali, O. , Coyle‐Thompson, C. , & Oppenheimer, S. B. (2004). Carbohydrate involvement in cellular interactions in sea urchin gastrulation. Acta Histochemica, 106(2), 97–106. 10.1016/j.acthis.2004.01.001 15147630

[gtc12934-bib-0024] Kimberly, E. L. , & Hardin, J. (1998). Bottle cells are required for the initiation of primary invagination in the sea urchin embryo. Developmental Biology, 204(1), 235–250. 10.1006/dbio.1998.9075 9851856

[gtc12934-bib-0025] Köhler, S. , Schmoller, K. M. , Crevenna, A. H. , & Bausch, A. R. (2012). Regulating contractility of the actomyosin cytoskeleton by pH. Cell Reports, 2(3), 433–439. 10.1016/j.celrep.2012.08.014 22999933 PMC3767110

[gtc12934-bib-0026] Kominami, T. , & Takata, H. (2004). Gastrulation in the sea urchin embryo: A model system for analyzing the morphogenesis of a monolayered epithelium. Development, Growth & Differentiation, 46(4), 309–326. 10.1111/j.1440-169x.2004.00755.x 15367199

[gtc12934-bib-0027] Levin, M. , Thorlin, T. , Robinson, K. R. , Nogi, T. , & Mercola, M. (2002). Asymmetries in H^+^/K^+^‐ATPase and cell membrane potentials comprise a very early step in left–right patterning. Cell, 111(1), 77–89. 10.1016/S0092-8674(02)00939-X 12372302

[gtc12934-bib-0028] Liu, D. , Awazu, A. , Sakuma, T. , Yamamoto, T. , & Sakamoto, N. (2019). Establishment of knockout adult sea urchins by using a CRISPR‐Cas9 system. Development, Growth & Differentiation, 61(6), 378–388. 10.1111/dgd.12624 31359433

[gtc12934-bib-0029] Martik, M. L. , & McClay, D. R. (2017). New insights from a high‐resolution look at gastrulation in the sea urchin, *Lytechinus variegatus* . Mechanisms of Development, 148, 3–10. 10.1016/j.mod.2017.06.005 28684256 PMC5705275

[gtc12934-bib-0030] McCain, E. R. , & McClay, D. R. (1994). The establishment of bilateral asymmetry in sea urchin embryos. Development, 120(2), 395–404. 10.1242/dev.120.2.395

[gtc12934-bib-0031] McClay, D. R. , Miranda, E. , & Feinberg, S. L. (2018). Neurogenesis in the sea urchin embryo is initiated uniquely in three domains. Development, 145(21), dev167742. 10.1242/dev.167742 30413529 PMC6240313

[gtc12934-bib-0032] Minokawa, T. , Rast, J. P. , Arenas‐Mena, C. , Franco, C. B. , & Davidson, E. H. (2004). Expression patterns of four different regulatory genes that function during sea urchin development. Gene Expression Patterns, 4(4), 449–456. 10.1016/j.modgep.2004.01.009 15183312

[gtc12934-bib-0033] Mizoguchi, H. (1999). Cell numbers in the gut of the embryo of the sea urchin *Hemicentrotus pulcherrimus* . Zoological Science, 16(2), 341–344. 10.2108/zsj.16.341

[gtc12934-bib-0050] Mullins, R. D. , & Hansen S. D. (2013). In vitro studies of actin filament and network dynamics. Current Opinion in Cell Biology, 25(1), 6–13. 10.1016/j.ceb.2012.11.007 23267766 PMC3578108

[gtc12934-bib-0034] Nájera, G. S. , & Weijer, C. J. (2020). Cellular processes driving gastrulation in the avian embryo. Mechanisms of Development, 163, 103624. 10.1016/j.mod.2020.103624 32562871 PMC7511600

[gtc12934-bib-0035] Nakajima, Y. , & Burke, R. D. (1996). The initial phase of gastrulation in sea urchins is accompanied by the formation of bottle cells. Developmental Biology, 179(2), 436–446. 10.1006/dbio.1996.0273 8903358

[gtc12934-bib-0036] Odell, G. M. , Oster, G. , Alberch, P. , & Burnside, B. (1981). The mechanical basis of morphogenesis: I. Epithelial folding and invagination. Developmental Biology, 85(2), 446–462. 10.1016/0012-1606(81)90276-1 7196351

[gtc12934-bib-0037] Oliveri, P. , & Davidson, E. H. (2004). Gene regulatory network controlling embryonic specification in the sea urchin. Current Opinion in Genetics & Development, 14(4), 351–360. 10.1016/j.gde.2004.06.004 15261650

[gtc12934-bib-0038] Pollard, T. D. , Blanchoin, L. , & Mullins, R. D. (2000). Molecular mechanisms controlling actin filament dynamics in nonmuscle cells. Annual Review of Biophysics and Biomolecular Structure, 29(1), 545–576. 10.1146/annurev.biophys.29.1.545 10940259

[gtc12934-bib-0039] Pollard, T. D. , & Cooper, J. A. (2009). Actin, a central player in cell shape and movement. Science, 326(5957), 1208–1212. 10.1126/science.1175862 19965462 PMC3677050

[gtc12934-bib-0040] Remsburg, C. , Testa, M. , & Song, J. L. (2021). Rab35 Regulates skeletogenesis and gastrulation by facilitating actin remodeling and vesicular trafficking. Cells and Development, 165, 203660. 10.1016/j.cdev.2021.203660 33842922 PMC8028849

[gtc12934-bib-0041] Schatzberg, D. , Lawton, M. , Hadyniak, S. E. , Ross, E. J. , Carney, T. , Beane, W. S. , Levin, M. , & Bradham, C. A. (2015). H^+^/K^+^ ATPase activity is required for biomineralization in sea urchin embryos. Developmental Biology, 406(2), 259–270. 10.1016/j.ydbio.2015.08.014 26282894

[gtc12934-bib-0042] Shimeld, S. M. , & Levin, M. (2006). Evidence for the regulation of left–right asymmetry in *Ciona intestinalis* by ion flux. Developmental Dynamics, 235(6), 1543–1553. 10.1002/dvdy.20792 16586445

[gtc12934-bib-0043] Shindo, A. (2018). Models of convergent extension during morphogenesis. Wiley Interdisciplinary Reviews: Developmental Biology, 7(1), e293. 10.1002/wdev.293 PMC5763355 28906063

[gtc12934-bib-0044] Stower, M. J. , & Bertocchini, F. (2017). The evolution of amniote gastrulation: The blastopore‐primitive streak transition. Wiley Interdisciplinary Reviews: Developmental Biology, 6(2), e262. 10.1002/wdev.262 28177589

[gtc12934-bib-0045] Takemoto, A. , Miyamoto, T. , Simono, F. , Kurogi, N. , Shirae‐Kurabayashi, M. , Awazu, A. , Suzuki, K.‐i. T. , Yamamoto, T. , & Sakamoto, N. (2016). Cilia play a role in breaking left–right symmetry of the sea urchin embryo. Genes to Cells, 21(6), 568–578. 10.1111/gtc.12362 27028068

[gtc12934-bib-0046] Tamulonis, C. , Postma, M. , Marlow, H. Q. , Magie, C. R. , de Jong, J. , & Kaandorp, J. (2011). A cell‐based model of *Nematostella vectensis* gastrulation including bottle cell formation, invagination and zippering. Developmental Biology, 351(1), 217–228. 10.1016/j.ydbio.2010.10.017 20977902

[gtc12934-bib-0047] Warner, J. F. , & McClay, D. R. (2014). Left–right asymmetry in the sea urchin. Genesis, 52(6), 481–487. 10.1002/dvg.22752

[gtc12934-bib-0048] Whittaker, J. R. , & Meedel, T. H. (1989). Two histospecific enzyme expressions in the same cleavage‐arrested one‐celled ascidian embryos. Journal of Experimental Zoology, 250(2), 168–175. 10.1002/jez.1402500208 2738555

[gtc12934-bib-0049] Yaguchi, S. , Yaguchi, J. , Angerer, R. C. , Angerer, L. M. , & Burke, R. D. (2010). TGFβ signaling positions the ciliary band and patterns neurons in the sea urchin embryo. Developmental Biology, 347(1), 71–81. 10.1016/j.ydbio.2010.08.009 20709054 PMC2950233

